# Applying graph database technology for analyzing perturbed co-expression networks in cancer

**DOI:** 10.1093/database/baaa110

**Published:** 2020-12-11

**Authors:** Claire M Simpson, Florian Gnad

**Affiliations:** Department of Bioinformatics and Data Science, Cell Signaling Technology Inc., 3 Trask Lane, Danvers, MA 01923, USA; Department of Bioinformatics and Data Science, Cell Signaling Technology Inc., 3 Trask Lane, Danvers, MA 01923, USA

## Abstract

Graph representations provide an elegant solution to capture and analyze complex molecular mechanisms in the cell. Co-expression networks are undirected graph representations of transcriptional co-behavior indicating (co-)regulations, functional modules or even physical interactions between the corresponding gene products. The growing avalanche of available RNA sequencing (RNAseq) data fuels the construction of such networks, which are usually stored in relational databases like most other biological data. Inferring linkage by recursive multiple-join statements, however, is computationally expensive and complex to design in relational databases. In contrast, graph databases store and represent complex interconnected data as nodes, edges and properties, making it fast and intuitive to query and analyze relationships. While graph-based database technologies are on their way from a fringe domain to going mainstream, there are only a few studies reporting their application to biological data. We used the graph database management system Neo4j to store and analyze co-expression networks derived from RNAseq data from The Cancer Genome Atlas. Comparing co-expression in tumors versus healthy tissues in six cancer types revealed significant perturbation tracing back to erroneous or rewired gene regulation. Applying centrality, community detection and pathfinding graph algorithms uncovered the destruction or creation of central nodes, modules and relationships in co-expression networks of tumors. Given the speed, accuracy and straightforwardness of managing these densely connected networks, we conclude that graph databases are ready for entering the arena of biological data.

## Introduction

The biology of the living cell results from complex interactions within a seemingly endless universe of molecular entities (1). Network representations have been successfully applied to understand a fraction of these cellular relationships, including protein–protein interaction (2), metabolic interplay (3), gene regulation (4) and gene co-expression (5). In these networks, nodes represent biological entities such as genes or proteins, edges represent their relationships and associated weights reflect the strengths of relationships. Gene co-expression networks are widely studied biological networks attributed to the plethora of available gene expression data. Even in the pre-RNAseq era, the abundance of microarray data allowed the generation of co-expression networks in various contexts. Mutual information and correlation coefficients (including Pearson and Spearman correlation) are the most common co-expression measures. Several studies have systematically compared the two measures and came to the consensus that both measures perform well in constructing co-expression networks on the basis of pairwise relationships (6).

The resulting biological networks have been conventionally stored and analyzed in traditional relational databases such as MySQL and Oracle. Inferring relationships with recursive multiple-join statements on tables, however, is complex to design and computationally expensive (7). In contrast, the concept of graph databases is to store interconnected data as a graph structure, so that querying and analyzing relationships become more intuitive and efficient. In fact, in recent years, graph database technologies have become widely used in various industries to analyze social interactions, purchasing behavior, flight connections and other connected data. The biological community has not fully embraced the advantages of graph databases yet, but several researchers have started to adopt the technology for integrating and analyzing biological data (8). For example, Reactome (reactome.org) adopted the graph database technology Neo4j (neo4j.com) to enable efficient access to biomolecular pathway data (9). The adaption of this technology reduced the average query time by 93%. Hetionet (neo4j.het.io) also adopted Neo4j to model drug efficacy based on hundreds of treatments and connected compounds, diseases, pathways, side effects, symptoms and other related data (10). The EpiGeNet framework used Neo4j to store and query conditional relationships between genetic and epigenetic events at different stages of colorectal oncogenesis (11). These and a few other examples suggest that well-established graph databases such as Neo4j are indeed ideal to capture and explore biological models. Notably, there are several other database technologies besides Neo4j. For example, RDF (Resource Description Framework) is a semantic graph database technology. It was established as a W3C standard for data exchange in the web representing data as a graph. Using Uniform Resource Identifiers standards, RDF is well suited to support ontologies and compare different datasets, which is, however, not the focus of our study.

In this study, we used RNA-seq data from The Cancer Genome Atlas (TCGA) to generate gene co-expression networks in several cancer types and the corresponding healthy tissues. We stored the resulting networks in Neo4j graph databases and applied centrality, community detection and pathfinding graph algorithms to detect genes, relationships and modules significantly altered in cancer networks. Our study confirms the advantage of using graph database technologies for storing and analyzing complex connected biological data.

## Materials and methods

### Generating co-expression matrices

For each sample, fragments per kilobase of transcript per million mapped reads (FPKM) data as a measure for gene expression were collected for the following cancer types from TCGA (portal.gdc.cancer.gov): BRCA (Breast Invasive Carcinoma) (112 samples) (12), KIRC (Kidney Renal Clear Cell Carcinoma) (49 samples) (13), LUAD (Lung Adenocarcinoma) (57 samples) (14), LUSC (Lung Squamous Cell Carcinoma) (49 samples) (15), PRAD (Prostate Adenocarcinoma) (52 samples) (16) and THCA (Thyroid Cancer) (58 samples) (17). For each cancer type, two co-expression matrices were calculated using FPKM data: one ‘normal’ matrix based on expression in healthy tissues and one ‘tumor’ matrix based on expression in the corresponding tumors. We limited our analysis to patients with available expression data for both normal and tumor. Co-expression matrices reflecting pairwise relationships between all 19,246 human genes were generated using the Weighted Gene Co-expression Network Analysis package (18) in R (r-project.org). Spearman coefficients were used as a measure for co-expression because of a higher outlier resistance compared with Pearson coefficients (Supplementary Figure 1). Correlations with Spearman coefficients above 0.99 were discarded, as all Spearman coefficients in this range resulted from near-zero expression of both genes in nearly all patients. Following the concept of soft thresholding, coefficients were raised to the sixth power and retained if they were above 0.25, yielding a scale-free topology of the networks (Supplementary Figure 2).

### Constructing and analyzing gene co-expression networks in Neo4j

The resulting co-expression matrices were used to create networks for each cancer type in Neo4j Desktop [Version 1.2.1 (1.2.1.1529)]. The Neo4j Desktop software allows the creation of graph database instances by uploading comma-separated value files containing nodes and edges information. A node was created for each gene in the dataset and labeled with its gene name, description and the corresponding UniProt ID (uniprot.org) (19). Edges were created between nodes to reflect the co-expression between the corresponding gene pair. Associated correlation coefficients were used as weights on these edges. Correlations from both normal and tumor data were included, forming two subnetworks within the same graph for each cancer type. The Cypher script used to load the database into Neo4j can be found in a github repository (https://github.com/clairesimpson95/cypher_coexp).

The Neo4j Algorithms package was used to apply the Pagerank, Louvain community detection and Dijkstra graph algorithms in each cancer type–specific network using the algorithms provided by Neo4j in the Cypher language. For labeling transcription factors, epigenetic regulators and oncogenes in the networks, we used annotations from Gene Ontology (geneontology.org) (20), a curated set of epigenetic regulators (5) and genes with hotspot mutations (21). Using all gene set collections from the Molecular Signatures Database (MSigDB) (22), gene set enrichment analysis was performed on communities comprising 50 or more genes. Additional annotation of specific genes was derived from UniProt and PhosphoSitePlus (phosphosite.org) (23). Graphs were generated in R using the packages ggplot2 (24), ggpubr (25), reshape2 (26) and dplyr (27).

All computations were performed locally on a MacBook Pro running macOS Mohave (Version 10.14.6) with a 2.3 HGz Intel Core i5 processor and 8 GB of memory.

## Results and discussion

### Building co-expression networks in Neo4j reveals substantial loss of gene regulation in cancer

To construct co-expression networks in healthy (normal) and tumor tissues, we calculated the degree of co-expression between each possible pair of genes across samples of the same tissue type (see the section “Materials and methods”). This resulted in two 19 246 × 19 246 correlation matrices (one for normal, one for tumor) for each of the six included tissue types. To translate each matrix into a network, a gene pair with significant co-expression was represented as two nodes and one connecting edge. Genes with no significant correlations were not included in the graph or in the total number of nodes. For each cancer type, we stored the corresponding co-expression networks of both healthy tissues and tumors in a Neo4j graph database instance (Methods).

**Figure 1.**
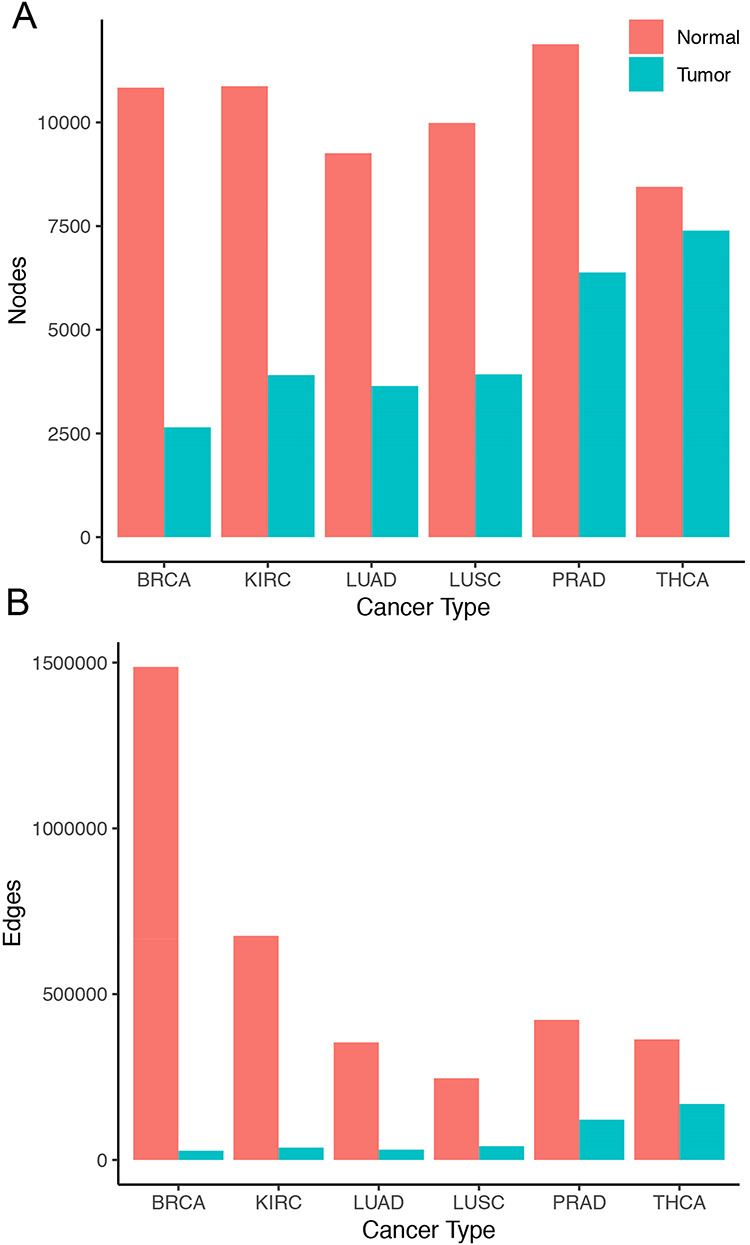
Size of co-expression networks. The bar charts show the total number of (A) nodes and (B) edges in the normal (red) and tumor (blue) co-expression graphs.

**Figure 2.**
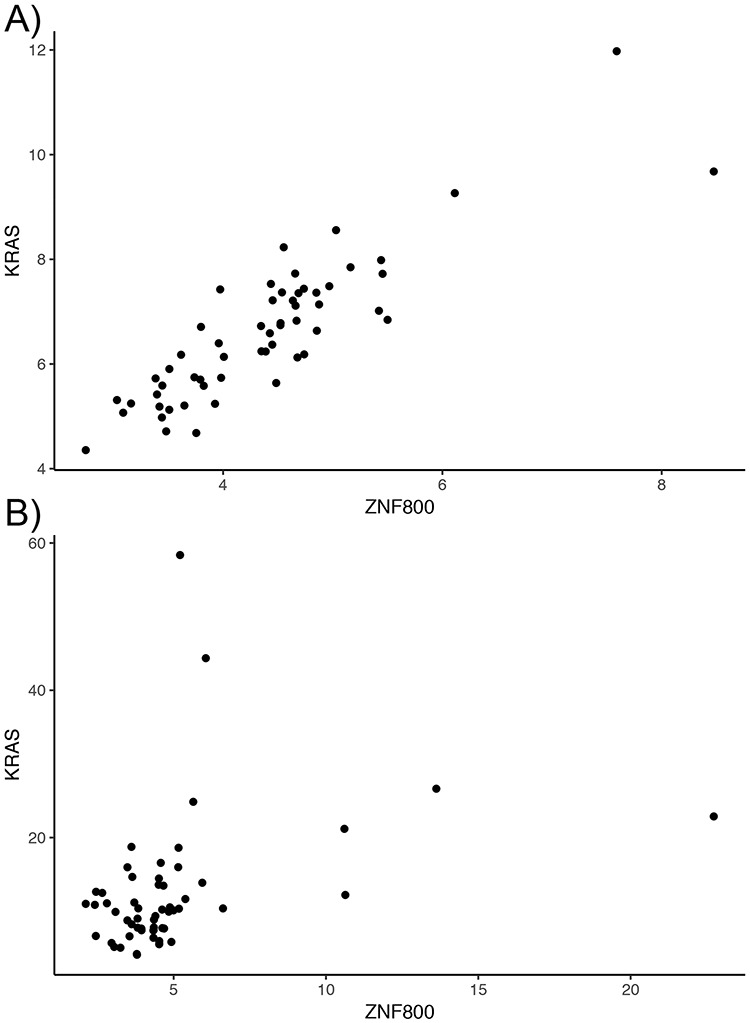
Lost co-expression in tumors. The dot plots illustrate the expression (measured as FPKM) of KRAS and ZNF800 in (A) healthy lungs and (B) lung adenocarcinoma. Each dot reflects the expression of the two genes in one sample.

Overall, tumor co-expression networks were significantly smaller than their normal counterpart co-expression networks (Figure 1). They contained 1.1-fold (THCA) to 4.2-fold (BRCA) fewer nodes and 2.2-fold (THCA) to 50.0-fold (BRCA) fewer edges. For example, the co-expression network of healthy lung tissues comprised 9257 nodes and 354 434 edges, while the network of the corresponding lung adenocarcinomas contained 3643 nodes and 31 019 edges. Overall, we identified 5676 co-expression relationships between transcription factors and oncogenes [defined by the presence of hotspot mutations (21)] lost in lung tumors. For example, we detected the co-expression between KRAS and ZNF800 (encoding zinc finger protein 800) in the lung normal network but not in the lung adenocarcinoma network (Figure 2). Concordantly, 1271 edges between epigenetic regulators and oncogenes were lost in tumor networks, such as co-expression between BRAF and PRDM2 (Supplementary Figure 3). Lost co-expression traced back to increased or decreased gene expression changes in either the epigenetic regulator or the oncogene.

The significant loss of nodes and edges in the tumor networks compared with their normal counterparts, which was also observed in a previous gene expression study (28), suggests massively perturbed coordination of gene expression in tumors. Moreover, tumor heterogeneity might further magnify the observed decrease in co-expression, and future single-cell experiments would be useful in understanding how strongly tumor heterogeneity might affect the results observed here.

### Applying centrality algorithms uncovers tumor-specific gene expression programs

**Figure 3.**
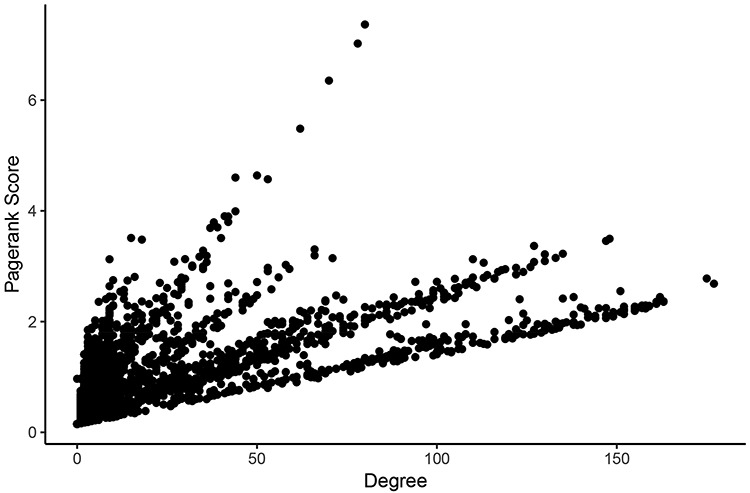
Comparison of measures of centrality in the LUAD tumor network. In the normal lung co-expression network, seven of the 10 nodes with the highest Pagerank scores were zinc finger transcription factors (Table 1). The other three genes encode trinucleotide repeat-containing gene 6A protein (TNRC6A), transmembrane protein-encoding gene KIAA1109 and nuclear mitotic apparatus protein 1 (NuMA-1).

For determining the most central nodes in each network potentially reflecting master regulators (such as epigenetic regulators or transcription factors) of the expression of many genes, we applied Google’s ‘Pagerank score’ algorithm and calculated the degree of each node (29). Both scoring algorithms take the number of edges of each node into account. The Pagerank score also includes the edges’ weights and the scores of neighboring nodes in its calculations. In each network, nodes were labeled with their Pagerank scores and degrees in healthy tissues and tumors. Calculating all scores within one Neo4j graph database instance for a given network took an average of 261 milliseconds. These scores can be found in Supplementary Tables 1–6. Pagerank score and degree were significantly correlated in all networks (Figure 3).

**Table 1.** The 10 genes with the highest Pagerank scores in the LUAD normal subnetwork

Name	Uniprot	Pagerank (normal)	Pagerank (tumor)	Degree (normal)	Degree (tumor)	Pathway
TNRC6A	Q8NDV7	4.426214	0.652741	653	4	Wnt signaling pathway
KIAA1109	Q2LD37	4.195813	0.718378	578	4	
ZNF397	Q8NF99	4.097128	0.9667225	602	1	
ZBTB37	Q5TC79	4.084897	0.596352	608	3	
ZNF662	Q6ZS27	4.066562	0.15	601	0	Generic transcription pathway
ZNF326	Q5BKZ1	4.060034	0.15	627	0	
NUMA1	Q14980	4.059286	0.15	276	0	Recruitment of NuMA to mitotic centrosomes, mitotic prophase
ZSCAN30	Q86W11	4.043646	0.15	599	0	
ZNF638	Q14966	4.041623	2.423036	615	22	Transcriptional regulation of white adipocyte differentiation
ZNF621	Q6ZSS3	4.036684	0.15	628	0	Generic transcription pathway

In the normal lung co-expression network, seven of the 10 nodes with the highest Pagerank scores were zinc finger transcription factors (Table 1). The other three genes encode trinucleotide repeat-containing gene 6A protein (TNRC6A), transmembrane protein-encoding gene KIAA1109 and nuclear mitotic apparatus protein 1 (NuMA-1). In fact, TNRC6A showed the highest Pagerank score in the normal lung network. The RNA levels of 653 genes correlated with the expression of TNRC6A in lung. The centrality of TNRC6A is consistent with its function as central organizer for RNA-mediated control of transcription (30). The second highest scoring gene was transmembrane protein-encoding gene KIAA1109. According to UniProt, the corresponding protein has been associated with endosomal trafficking and recycling, regulation of phagocytosis, regulation of cell growth and other biological processes. A direct link to master regulation of gene expression, however, has not been described to the best of our knowledge. Finally, NUMA1, playing a significant role in the formation and maintenance of the spindle poles and chromosome alignment and segregation during mitotic division (31), was the third non-zinc finger gene in the top 10 of the central nodes.

Collectively, nodes tended to have higher Pagerank scores and degrees in the normal networks compared with tumor networks (Supplementary Figures 4 and 5). The Pagerank scores of 78% of all nodes including the 10 top scoring nodes were lower in the lung adenocarcinoma network compared with the healthy lung network. Six percent of all nodes showed higher Pagerank scores but lower degrees in the tumor network, indicating a relative but not absolute increase in centrality. Table 2 lists the top 10 nodes with respect to PageRank scores falling into this category. The associated proteins of these 10 nodes are involved in a variety of fundamental cellular processes including response to DNA damage (RIF1, RNF169), epigenetic regulation (BPTF, KMT2A), splicing (SCAF11) or cell death (BIRC6). At least four proteins are directly involved in protein ubiquitination including USP34, BIRC6, UBXN7 and RNF169.

**Table 2.** The 10 genes with the highest Pagerank scores in the lung adenocarcinoma network

Name	Uniprot	Pagerank (normal)	Pagerank (tumor)	Degree (normal)	Degree (tumor)	Pathway
RIF1	Q5UIP0	2.503803	7.365182	325	80	Nonhomologous end-joining (NHEJ)
USP34	Q70CQ2	2.5970565	7.021621	328	78	TCF dependent signaling in response to WNT, Ub-specific processing proteases
BPTF	Q12830	3.906405	6.351855	563	70	
BIRC6	Q9NR09	2.158023	5.483818	281	62	
KMT2A	Q03164	3.977516	4.639488	535	50	RUNX1 regulates transcription of genes involved in differentiation of HSCs
UBXN7	O94888	3.775539	4.600906	514	44	Neddylation
ASXL2	Q76L83	2.398658	4.571309	305	53	UCH proteinases
RNF169	Q8NCN4	0.8521765	3.992094	79	44	Ubl conjugation pathway
ALMS1	Q8TCU4	1.4537725	3.903609	147	41	AURKA activation by TPX2, recruitment of mitotic centrosome proteins and complexes
SCAF11	Q99590	3.153033	3.899333	431	42	

**Figure 4.**
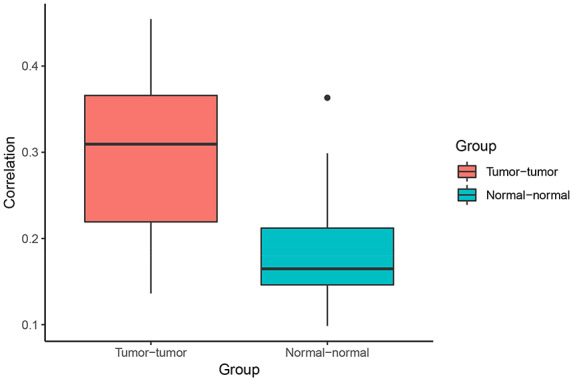
Correlation of Pagerank scores between tumor networks (red) and normal networks (blue). On average, the correlation between Pagerank scores in tumor networks was higher than the correlation between Pagerank scores in normal networks (excluding the two normal lung tissue networks). Higher PageRank scores indicate higher centrality in the network.

Overall, 36 genes showed an increase in degree from normal to tumor networks in at least five of the six cancer types (Supplementary Table 7). Gene set enrichment analysis revealed that these 36 genes are most significantly enriched for genes involved in epithelial mesenchymal transition (including THY1, MMP2, VCAN, COMP, PRRX1 and COL12A1) and the immune system (including GRAP2, SAA1, GBP1, CIITA, CD80, KIF23, IRF8, TAP1 and GBP4) (Supplementary Table 8).

When comparing centrality between co-expression networks, we found that Pagerank scores showed higher Pearson correlations between tumor networks than between normal networks. The average Pearson correlation for PageRank scores between two tumor networks was 0.30, whereas the average correlation between two normal networks was 0.21 (*P* = 0.003 using t-test excluding the comparison between the two normal lung networks) (Figure 4). This suggests that gene expression programs are relatively similar across different cancer types. In comparison, expression programs are quite distinct between healthy tissue types.

### Community detection in Neo4j reveals involvement of central tumor subnetworks involved in immune system and replication

To identify highly connected subnetworks (communities) within each network, we applied the Louvain algorithm (32) in Neo4j. The algorithm finished in under a second for all graphs with less than 5000 nodes. It took three seconds for the largest network, the normal breast network, containing 11 884 nodes.

**Figure 5.**
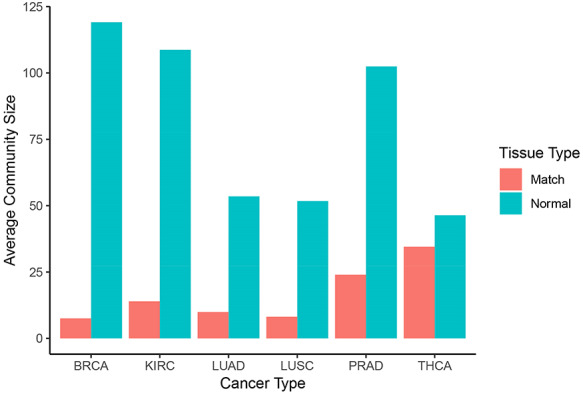
Community sizes across cancer and tissue types. Number of genes per community resulting from applying the Louvain algorithm on each graph in Neo4j (blue: normal, red: matched tumor).

**Figure 6.**
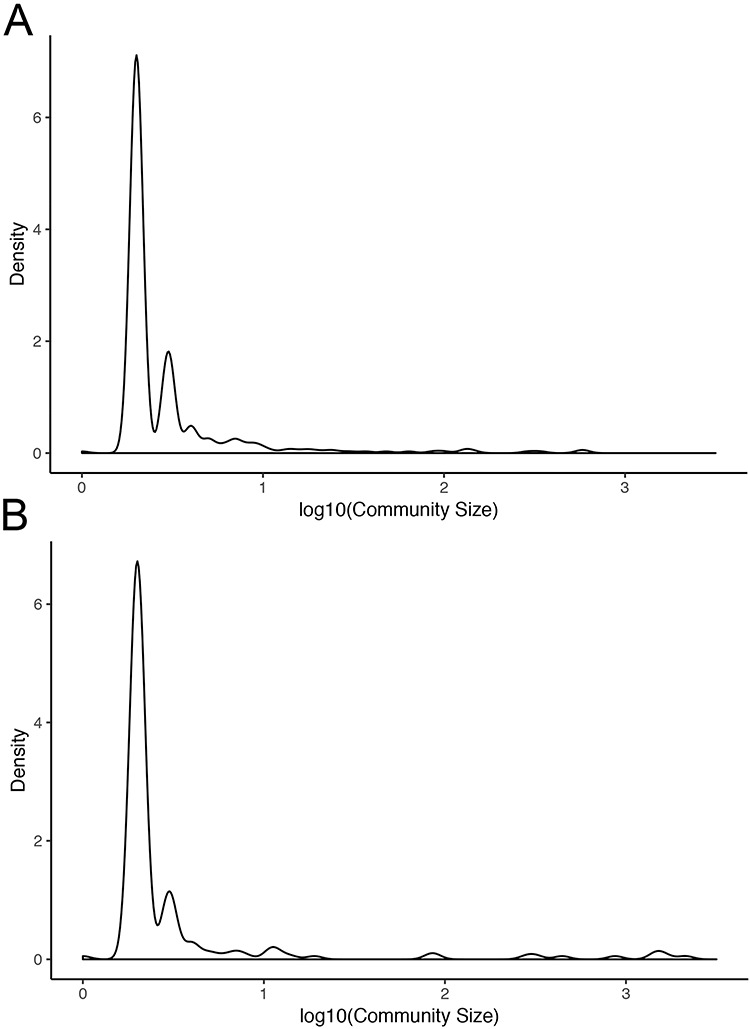
Community sizes in LUAD networks. Density plots of community size in LUAD (A) tumor and (B) normal graphs.

Normal networks had fewer communities but more members in each community compared with tumor networks. The average number of genes per community in normal networks ranged from 46.41 (THCA) to 119.09 (BRCA) and from 8.16 (LUSC) to 34.54 (THCA) in the tumor networks (Figure 5). The number of communities ranged from 91 (BRCA) to 193 (LUSC) in normal networks and from 214 (THCA) to 481 (LUSC) in tumor networks. For instance, in LUAD, there were 173 communities in the normal network and 367 communities in the tumor network. While 91% and 94% communities contained less than 10 nodes in the normal and in the tumor network, respectively, the range in community size was greatest in the normal network. In the LUAD graph, the largest normal community contained 2135 nodes, while the largest tumor community contained 600 nodes (Figure 6).

**Figure 7.**
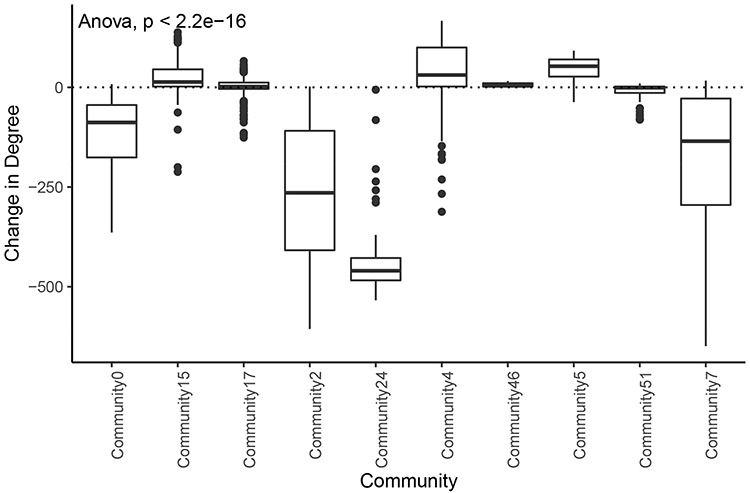
Change in degree across large communities in LUAD. Difference of degree between tumor and normal samples for each tumor community larger than 50 genes.

We also found that communities had significantly different distributions of degree change in the tumor network compared with the normal network (Figure 7). This implies that different communities and associated biological processes are affected to varying degrees by the transition to cancer status. Overall, 76% (84/110) of the nodes with an increase in degree of greater than 100 were in the tumor subnetwork’s community 4. Gene set enrichment analysis revealed that this community was enriched for genes involved in several cell cycle processes (Supplementary Table 9). Similarly, 124 of 129 genes in community 5 showed increased degrees, and a significant proportion of these genes are involved in the immune system. The tumor subnetwork’s community 15, whose nodes also had on average a higher degree in the tumor than in the normal subnetworks, was also enriched for immune response genes. Consistent with LUAD, communities of other tumor networks, which showed increased degrees, were also functionally enriched for immune system processes or DNA replication.

**Figure 8.**
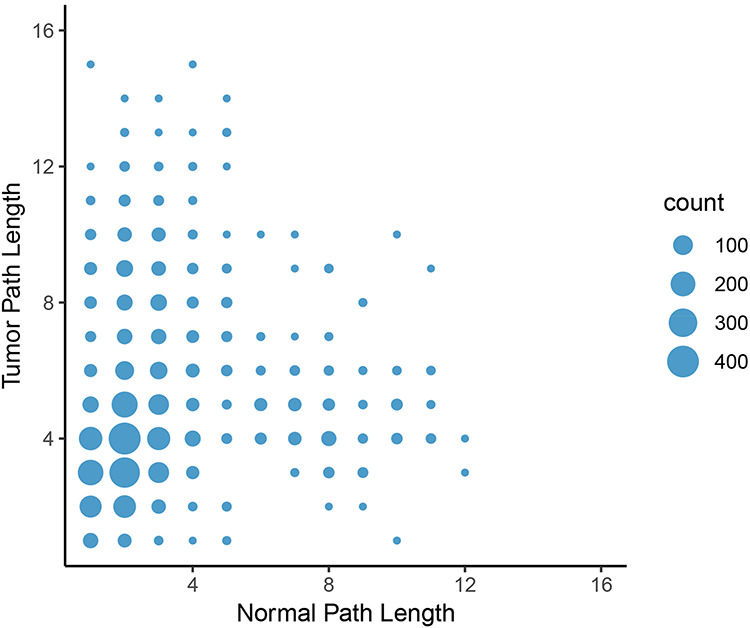
Change in path length between LUAD normal and tumor networks. On average, the path length between an epigenetic regulator and an oncogene increased from the normal graph to the tumor graph, with the most common change in path length from two to four genes.

Overall, the application of community detection in Neo4j revealed several commonalities between communities of different cancer types with respect to changes of degree, size distributions and functional associations, confirming a common tumor-specific reprogramming of the transcriptional system.

### Shortest path algorithm uncovers interrupted connections between oncogenes and epigenetic regulators

To calculate the shortest paths between nodes, we applied Neo4j’s implementation of Dijkstra’s algorithm. We focused on shortest paths between all pairs of oncogenes and epigenetic regulators found in the graphs to elucidate potential differences in regulation of oncogene expression by epigenetic programs. A shorter path between two genes might indicate that they are more tightly co-regulated and intermediate controls are lost. In contrast, a longer path in tumor tissue might indicate that direct regulation of gene expression is lost and additional controls are involved. Consistent with the lower number of nodes and loss of centrality in tumor graphs compared to normal graphs, the average length of a shortest path between the proteins in these groups increased from 3.88 in the normal graph to 4.76 in the tumor graph in the LUAD graph. Furthermore, the number of shortest paths of infinite length increased from 1320 in the normal set to 61 995 in the tumor set, indicating that no path between these pairs was possible. In other words, the link between the expression of oncogene and potentially connected epigenetic regulators were broken in the LUAD tumor network. The same trend was observed in the other cancer types. The most frequent change in path length (where both paths have finite length) was from two in the normal graph to four in the tumor graph (Figure 8). These results further indicate a systemic loss of gene expression coordination in tumor cells.

## Conclusion

In our study, the graph database management system Neo4j has proven to be an efficient solution to study co-expression networks in healthy tissues and in tumors. Associated tools allowed for quick creation, maintenance and analysis of the connected data. We found that the majority of gene co-expressions are lost in cancer. However, compared to healthy tissues, tumor co-expression networks are relatively similar to each other, pointing to tumor-specific expression programs. We also uncovered several tumor-specific master nodes, communities and paths. Overall, our study demonstrates the power of graph databases to understand complex biological networks, and we believe that this technology is ready to become mainstream for bioinformatics.

## Supplementary Material

baaa110_Supp
