# Supplementary figures and images for: Applying graph database technology for analyzing perturbed co-expression networks in cancer
Source: Database (Oxford). 2020 Dec 11;2020:baaa110. doi: 10.1093/database/baaa110 (PMC7731929; doi:10.1093/database/baaa110)

A

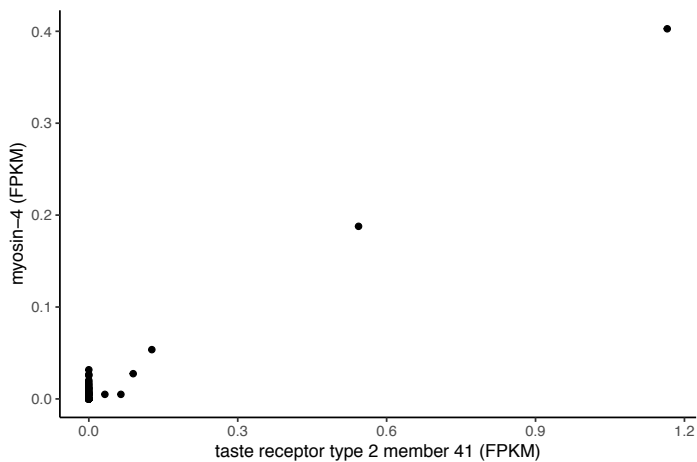

B

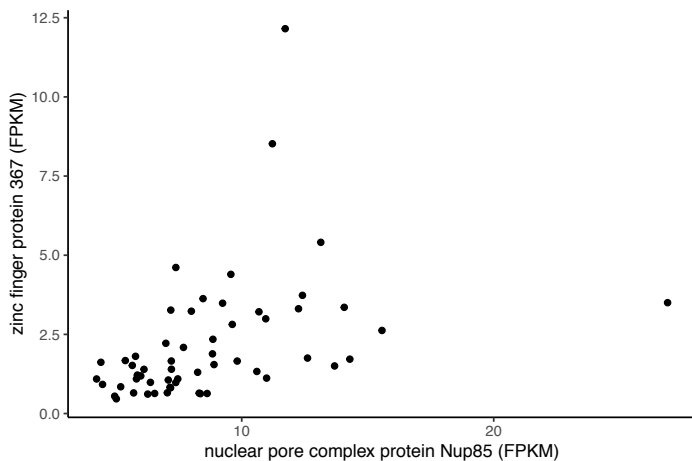

Supplement: baaa110_Supp [file baaa110_supp.zip › suppl_data/Suppl Fig 1.pdf]

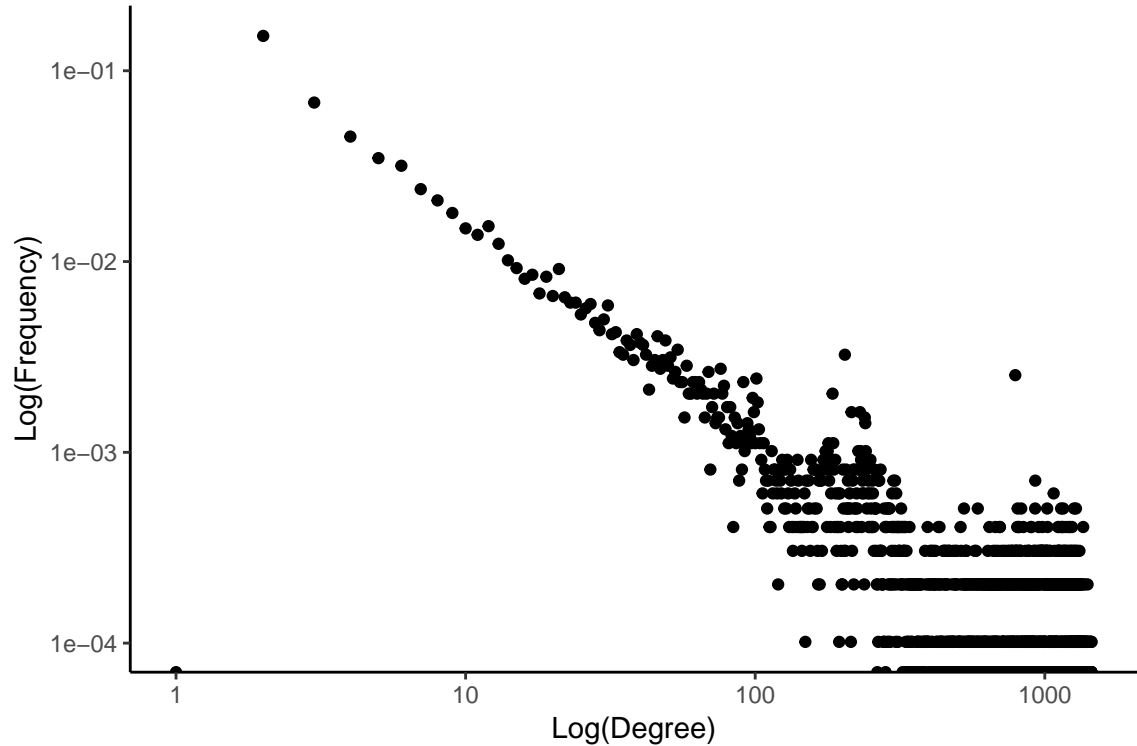

Supplement: baaa110_Supp [file baaa110_supp.zip › suppl_data/Suppl Fig 2.pdf]

A

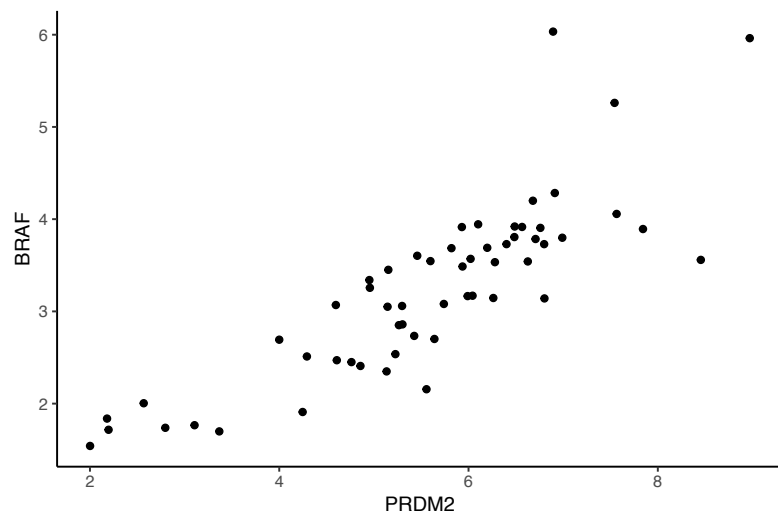

B

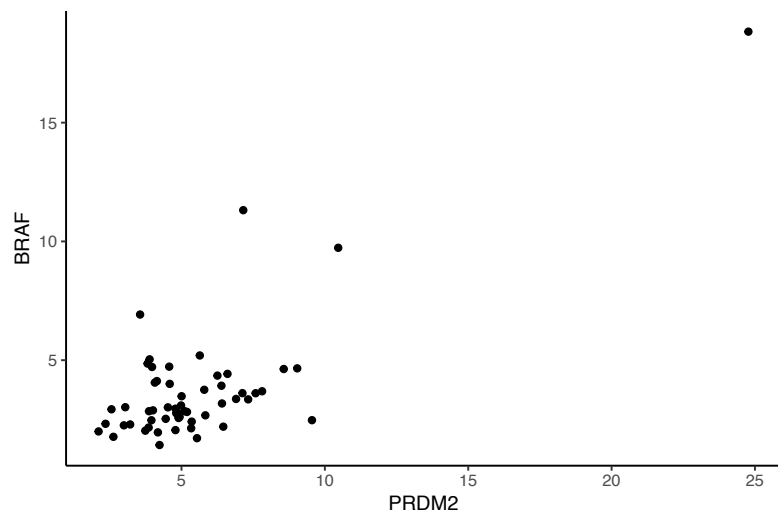

Supplement: baaa110_Supp [file baaa110_supp.zip › suppl_data/Suppl Fig 3.pdf]

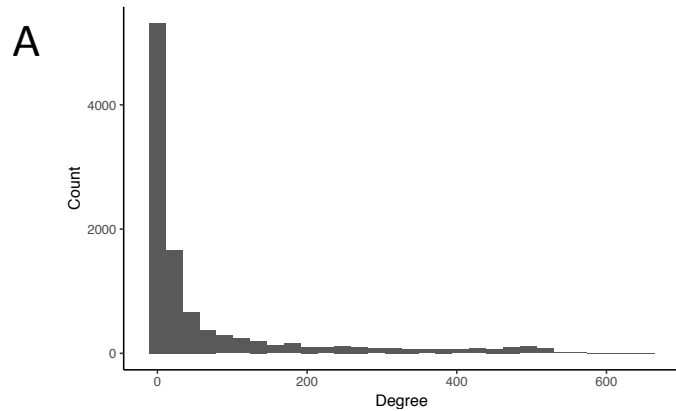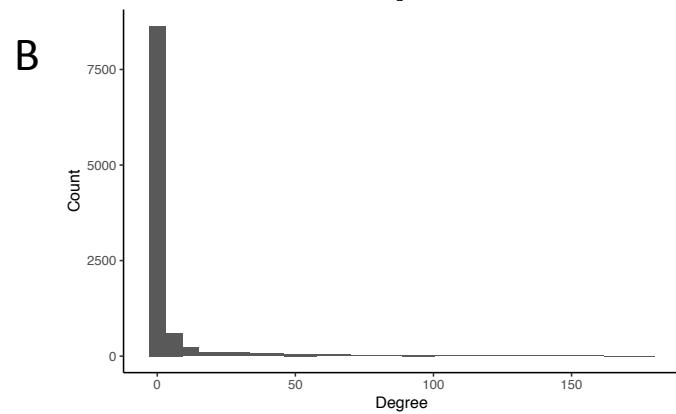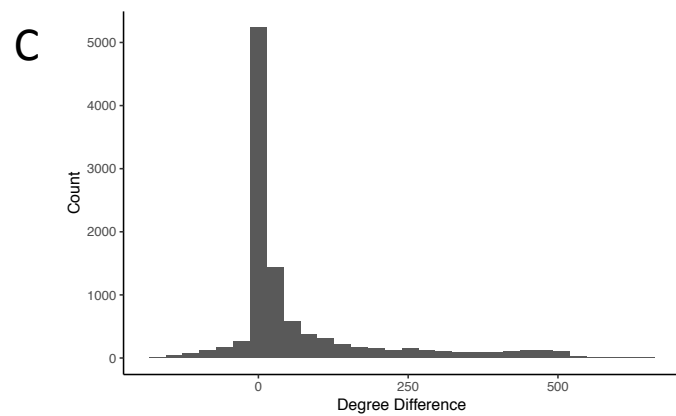

Supplement: baaa110_Supp [file baaa110_supp.zip › suppl_data/Suppl Fig 4.pdf]

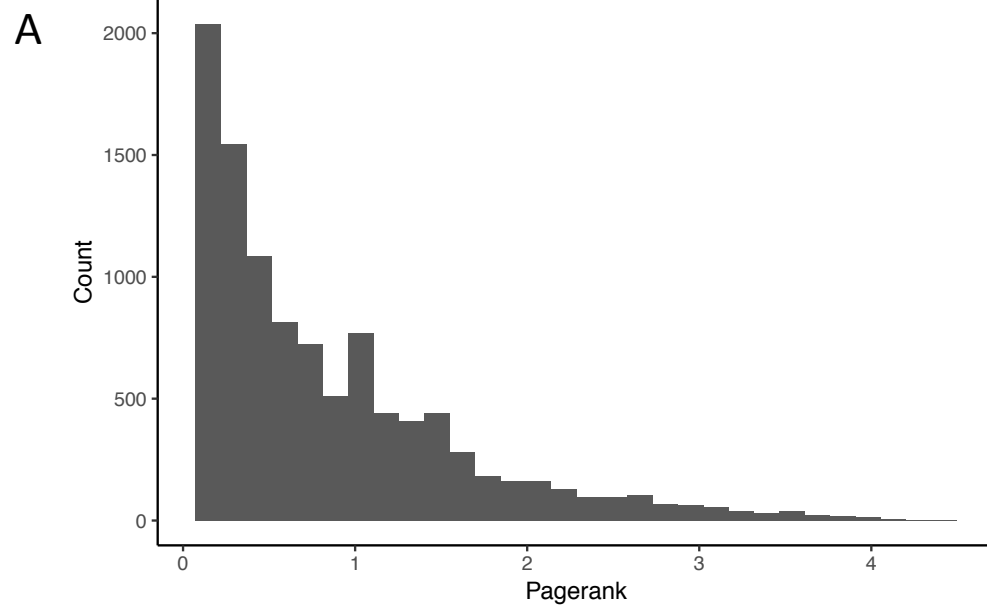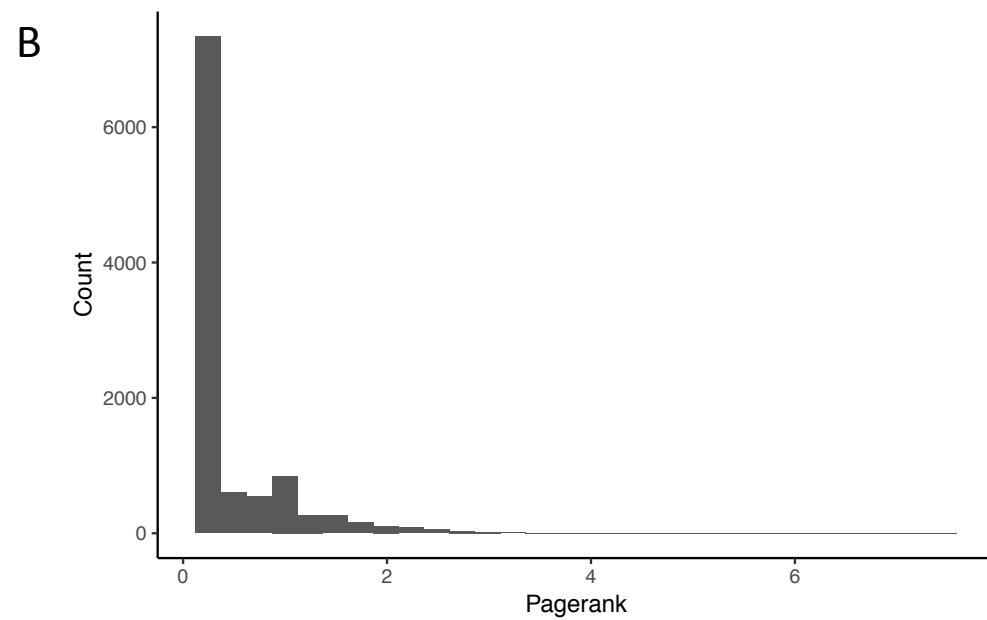

Supplement: baaa110_Supp [file baaa110_supp.zip › suppl_data/Suppl Fig 5.pdf]
